# The effect of lactic acid bacteria and co‐culture on structural, rheological, and textural profile of corn dough

**DOI:** 10.1002/fsn3.2666

**Published:** 2021-11-30

**Authors:** Sanabil Yaqoob, Huimin Liu, Meihong Liu, Mingzhu Zheng, Kanza Aziz Awan, Dan Cai, Jingsheng Liu

**Affiliations:** ^1^ College of Food Science and Engineering Jilin Agricultural University Changchun China; ^2^ National Engineering Laboratory for Wheat and Corn Deep Processing Changchun China; ^3^ Department of Food Science and Technology Faculty of Life Sciences University of Central Punjab Lahore Pakistan

**Keywords:** corn dough, lactic acid bacteria, rheology, texture

## Abstract

This study is aimed at assessing the effect of lactic acid bacteria (LAB) on corn flour using dynamic characterization methods including RVA, TPA, Rheometer, *SEM*, and DSC along with co‐culture technique in order to enhance its applicability by evaluating the variations in rheological, textural, morphological, thermal, and structural properties. Our findings suggested that bacterial incorporation both individually and in combination (co‐culture) revealed an improved corn dough profile with better properties. *SEM* showed irregular shape of particles having more grooves, indentations, and cracks. RVA demonstrated different pasting behavior on the dough. Bacterial inoculation in flour attributed to increase the TO (68.61–71.18), TP (73.74–78.42), TC (78.78–85.36), melting temperature (10.17–15.19), and ΔH (2.72–5.40). The hardness of corn was found approximately 75% of native dough. In treated corn, an increase was noted in both loss and storage modulus in correspondence with changes in the starch configuration and leaching of constituents. The results from DSC presented an increased melting temperature range and gelatinization enthalpy owing to bacterial treatment accredited to diversified morphological characteristics. The outcomes concluded in demonstration of a novel influence on structural, thermal, morphological, and rheological capabilities and capacities of corn dough. Lactic acid bacteria hydrolyzed part of the corn and flour had smaller, irregularly shaped particles with more holes in them, resulting in a reduced water retaining capacity. Textural, thermal, and pasting profile has also been improved due to degradation of macromolecules. Furthermore, the insight alterations induce various changes leading to improved corn flour. It may also develop the associations about the upright insurgence in the corn dough profile and its potential usage in industry and homes.

## Graphical abstract

Assessing the effect of *L. casei*, *L. plantarum* and *L. fermentum* on corn flour using dynamic characterization methods including RVA, TPA, Rheometer, *SEM*, DSC along with co‐culture technique in order to enhance its applicability by evaluating the variations in rheological, textural, morphological, thermal and structural properties.

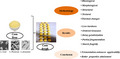

## 1 INTRODUCTION

During the last epoch, microbial biotechnology has attained phenomenal attention due to its huge role in production of commercially valuable products. Among them, lactic acid bacteria (LAB) are of particular interest owing to its widespread applications especially in food industry such as yogurt and cheese. It can also be applied as emulsifier, improving agents, and esters due to its hygroscopic nature (Castillo Martinez et al., 2013). Lactic acid fermentation can provide a lot of benefits, such as lower energy consumption, cheap renewable substrate, and production of pure lactic acid. It is also considered as a precursor of several large and small compounds (Abdel‐Rahman et al., 2013). Food and drug administration announced lactic acid and its salts as generally recognized as safe (GRAS) (Arshadi et al., 2016). Co‐ culture is a recent fermentation technique wherein two or more populations are cultured together to stimulate growth and to produce a determined product (Eş et al., 2017). Microbial technology is very delicate branch of science because of the production of large amount of by‐products such as ethanol and acetic acid which required further separation and purification and ultimately increase overall cost. Mostly the homofermentative LAB are unable to covert sugars into lactic acid while heterofermentative LAB can easily convert sugars into lactic acid. These heterofermentative strains also produce a lot of ethanol and acetic acid. The third most pronounced form is facultative heterofermentative, they use glucose to produce lactic acid via Embden‐Meyerhof pathway. Therefore, co‐culture that can outcompete with other bacteria or strain will reduce byproduct accumulation. Zhang and Vadlani, (Zhang & Vadlani, 2015) used *L. plantarum* and *L. brevis* as co‐culture and their results concluded high yield while lower by‐product accumulation.

Cereals are the most extensively used crops of the world and corn ranked third among them. Corn is full of nutrients and wisely used in food production or direct consumption. Only 5% of the produced corn is used for human consumption while rest of it (95%) is transformed into fodder and alcohol. Owing to certain limitations like low shear and thermal resistance, mucin deficiency, higher retrogradation and thermal decomposition, which limits its functional properties in industry. However, several modifications such as esterification, acidic hydrolysis, carbonation, etherification, polymerization, extrusion, addition of gums, and enzymolysis have been used to improve the applicability of corn flour (Reyes et al., 2016). The chemical modification is easy to achieve but it worsens the taste and also unhealthy for humans. Extrusion is a prevalent technique, but its cost is very high. Biotechnological modification changes the molecular structure and gives high specificity by improving the corn flour (Uthumporn et al., 2012).

In this study, LAB (*L. casei, L. plantarum, L. fermentum*) were used to improve corn flour properties. The objective of the present investigation was to characterize rheological, morphological, structural, textural, and thermal changes in corn flour due to the inoculation of multiple bacteria (coculture) and single bacteria. The obtained outcomes of the study may provide innovative insights about the corn granules and its deterioration, thereby providing a comprehensive data to improve the quality of a final product.

## 2 MATERIALS AND METHODS

For this study, corn grains (Jingke 968) were obtained from Jilin Agricultural University, China. Milling of the grains was carried out using laboratory miller (TAISITE, FW100) the flour was sieved and the fraction retained (0.180 mm) was used. *Lactobacillus casei (L. casei), Lactobacillus plantarum (L. plantarum) and Lactobacillus fermentum (L. fermentum)* were propagated on MRS medium at 37°C and stored at 4°C. Active dry yeast (Angel brand), sugar and salt were purchased from local market. All other chemicals were of analytical grade.

### 2.1 Fermentation conditions

Isolated *L. casei, L. plantarum, and L. fermentum* were grown in MRS medium (liquid) in an incubator (HZQ‐F160) to a bacterial concentration of 10^7^–10^8^ cfu/mL. Twenty percent bacterial suspension was added in corn flour and placed in incubator for 1–5 days at 37°C. Dough was then prepared by mixing all ingredients thoroughly Table 1.

**TABLE 1:** Amount of *L. Casei, L. Plantarum, and L. Fermentum* for the fermentation of corn flour

Treatment	*L. casei*	*L. fermentum*	*L. plantarum*
CF	‐	‐	‐
LC	200 mL	‐	‐
LF	‐	200 mL	‐
LP	‐	‐	200 mL
LC‐LF	100 mL	100 mL	‐
LC‐LP	100 mL	‐	100 mL
LF‐LP	‐	100 mL	100 mL
LC‐LF‐LP	67 mL	67 mL	67 mL

Note

This respective value is for 100 g corn flour.

Simple corn flour (CF), *Lactobacillus casei‐*treated corn flour (LC), *Lactobacillus fermentum‐*treated corn flour (LF), *Lactobacillus plantarum‐*treated corn flour (LP), *Lactobacillus casei and Lactobacillus fermentum‐*treated corn flour (LC‐LF), *Lactobacillus casei* and *Lactobacillus plantarum‐*treated corn flour (LC‐LP), *Lactobacillus plantarum* and *Lactobacillus fermentum‐*treated corn flour (LF‐LP), *Lactobacillus casei*, *Lactobacillus plantarum, and Lactobacillus fermentum‐*treated corn flour (LC‐LF‐LP).

### 2.2 Pasting properties of flour

The pasting properties were ascertained by using Rapid Visco Analyzer (Perten) as per the procedure described by Wani (Wani et al., 2012). Three grams (3 g) of flour was added in 25 mL of water and placed on RVA cup. The average values for peak viscosity (PV), Trough viscosity (TV), Final viscosity (FV), breakdown, setback, and pasting temperature (PT) were obtained for each sample.

### 2.3 Micromorphology of corn

Scanning electron micrographs (Phenom^TM^) of each sample were obtained at magnifications of ×5000. Flour samples were fixed on the holders with double spread and gold layer was sputtered on it and then scanned in a vacuum of 5kv potential difference.

### 2.4 Textural analysis of corn flour

Fifty grams (50 g) of corn flour was kneaded with 50 mL of water in order to make a standard dough by giving 60 min of fermentation at room temperature. The textural analysis comprises hardness, adhesiveness, springiness, cohesiveness, chewiness, gumminess, and resilience were measured by a textural analyzer (TA.XT plus). Maximum force applied can be considered as hardness. The conditions were as follows: test distance 10 mm, probe P/0.5, and test velocity were 0.5 mm.

### 2.5 Rheological characterization of dough

Rheological properties were measured by using rheometer (Anton Paar, Modulus compact rheometer MCR‐302). Frequency sweep test was performed by adopting the following parameters: parallel plates (50 mm), a gap (1 mm), temperature 25°*C*. The sample was placed on the plate and excessive material was wiped off through the spatula. Silicone oil is added to the sample to avoid the evaporation and 10 min rest was given to equilibrate the stresses. First of all, the linear viscoelastic region was defined through a strain sweep test. Storage (G'), loss modulus (G"), and tangent delta (tan) were determined at constant shear strain and frequency range 0.1–10 Hz.

### 2.6 Thermal characterization of flour

Thermal properties of flour samples were analyzed by differential scanning calorimetry (DSC, TA Q 2000). Analysis conditions were as follows: 3 mg sample, 6 μL of distilled water, temperature 30–90°C at a constant rate of 10°C min^‐1^. An empty aluminum pan was used as a reference. The respective parameters were determined with the use of universal analysis 2000 software.

### 2.7 Statistical design

The obtained data for each parameter were subjected to appropriate statistical analysis through Statistical Package Origin‐Pro 8.5 software. The analysis was performed using completely randomized design (CRD) and the analysis of variance was applied to determine the level of significance.

## 3 RESULTS AND DISCUSSION

### 3.1 Textural analysis of corn flour

A remarkable variation in textural analysis between simple corn flour and bacterial treated corn flour were observed in this study (Table 2). Bacterial incorporation in corn flour improved the textural properties by reducing hardness and more cohesiveness, gumminess, chewiness, and springiness which enables easy processing of flour into dough. While LC, LF, and LC‐LP results in more hardness, which may be due to denaturation and gelation of proteins and loss of water. It is reported that the acid‐induced gelation due to LC and LF causes rigidity in flour and results in more hardness (Hu et al., 2007). Yang (Yang et al., 2017) worked on the fermented amylase‐rich fortified maize flour and found better textured fermented flour concluded less hardness, more cohesiveness, gumminess, and chewiness which is in accordance to this study. Oladeji (Oladeji et al., 2014) analyzed and developed carrot‐fortified fermented corn flour and concluded some favorable rheological attributes which is more compatible to produce a good product. Starch retrogradation is responsible for textural hardness and it is less pronounced in bacterial treated flour. The process involved may be gelation of amylose and recrystallization of amylopectin within gelatinized granules. Gelation involved a fast development via chain enlargement while amylopectin involved slow crystallinity. Therefore, textural increment with bacteria could be related with retrogradation phenomenon (Tao et al., 2016). Our previous studies of fermentation through yeast concluded better textural profile due to lower hardness, more cohesiveness, and gumminess which is compatible with the current studies (Sanabil et al.,^2019^).

**TABLE 2:** Effect of *L. Casei, L. Plantarum, and L. Fermentum* on the textural properties of corn flour dough

Treatment	Hardness (g)	Adhesiveness (g.sec)	Springiness (mm)	Cohesiveness	Gumminess (g)	Chewiness	Resilience
CF	87.89 ± 0.78^c^	−61.84 ± 0.48^e^	0.82 ± 0.06^b^	0.22 ± 0.01^c^	33.62 ± 0.40^d^	28.58 ± 0.43^d^	0.13 ± 0.02^a^
LC	94.83 ± 1.68^b^	−46.67 ± 0.39^c^	0.99 ± 0.04^a^	0.26 ± 0.05^bc^	25.45 ± 0.43^e^	25.34 ± 0.46^d^	0.12 ± 0.01^ab^
LF	107.02 ± 1.60^a^	−43.53 ± 0.41^b^	0.92 ± 0.07^ab^	0.34 ± 0.06^abc^	37.27 ± 0.56^b^	33.84 ± 0.60^e^	0.08 ± 0.01^ab^
LP	65.43 ± 1.37^f^	−39.72 ± 0.33^a^	0.90 ± 0.06^ab^	0.38 ± 0.07^abc^	23.41 ± 0.40^f^	21.47 ± 0.42^f^	0.10 ± 0.03^ab^
LC‐LF	71.33 ± 0.49^e^	−44.03 ± 0.42^b^	0.93 ± 0.05^ab^	0.47 ± 0.11^a^	33.92 ± 0.49^d^	31.99 ± 0.76^c^	0.07 ± 0.01^b^
LC‐LP	89.30 ± 1.81^c^	−96.15 ± 0.33^g^	0.88 ± 0.08^ab^	0.45 ± 0.13^ab^	40.23 ± 0.37^a^	35.96 ± 0.80^a^	0.07 ± 0.02^ab^
LF‐LP	80.19 ± 0.74^d^	−66.93 ± 0.73^f^	0.85 ± 0.07^ab^	0.48 ± 0.05^a^	35.73 ± 0.25^c^	31.08 ± 0.54^c^	0.09 ± 0.04^ab^
LC‐LF‐LP	71.74 ± 0.22^e^	−49.80 ± 1.17^d^	0.91 ± 0.06^ab^	0.46 ± 0.15^a^	37.26 ± 0.24^b^	34.43 ± 0.44^b^	0.08 ± 0.02^ab^

Note

Simple corn flour (CF), *Lactobacillus Casei‐*treated corn flour (LC), *Lactobacillus Fermentum‐*treated corn flour (LF), *Lactobacillus Plantarum‐*treated corn flour (LP), *Lactobacillus Casei and Lactobacillus Fermentum‐*treated corn flour (LC‐LF), *Lactobacillus Casei* and *Lactobacillus Plantarum‐*treated corn flour (LC‐LP), *Lactobacillus Fermentum* and *Lactobacillus Plantarum‐*treated corn flour (LF‐LP), *Lactobacillus Casei*, *Lactobacillus Fermentum,* and *Lactobacillus Plantarum‐*treated corn flour (LC‐LF‐LP).

a‐g: Means in the same columns with different letters were significantly different (*p* < .05) from each other.

### 3.2 Thermal characterization of flour

Results depicted in Table 3 showed the thermodynamic properties including the onset‐T_O_, peak‐T_P_, conclusion‐T_C_, melting temperature T_C_ ‐T_O,_ and gelatinization enthalpy ΔH of flour samples as determined by DSC. Native corn flour showed onset (T_O_), peak (T_P_), conclusion (T_C_), melting temperature (T_C_ ‐T_O_), and gelatinization enthalpy ΔH were 68.61°C, 73.74°C, 78.78°C, 10.17°C, and 2.72 J/g, which is in line with the reported values by Yangcheng (Yangcheng et al., 2013). On the other hand, bacterial incorporation in flour attributed to increase the T_O_ (68.61–71.18), T_P_ (73.74–78.42), T_C_ (78.78–85.36), melting temperature (10.17–15.19), and ΔH (2.72–5.40). The increasing transition temperatures and gelatinization enthalpy reflected more ordered granules and higher degree of crystallinity, which ultimately results in higher temperatures and leading to delayed gelatinization, which is in agreement with previous studies by Tao who concluded that the higher gelatinization temperature results in delayed gelatinization; ultimately, the granules become more resistant to be gelatinized (Tao et al., 2015). Kaur (Kaur et al., 2004) reported that phosphorus content, amylose to amylopectin ratio, degree of branching, molecular conformation, and its length directly influenced the gelatinization temperature and enthalpy.

**TABLE 3:** Effect of *L. Casei, L. Plantarum, and L. Fermentum* on the Thermodynamic parameters of corn flour dough

Treatment	T_O_ (^O^C)	T_P_ (^O^C)	T_C_ (^O^C)	T_C_ ‐T_O_ (^O^C)	ΔH (J/g)
CF	68.60 ± 0.07^d^	73.74 ± 0.13^f^	78.78 ± 0.11^c^	10.17 ± 0.03^c^	2.72 ± 0.17^f^
LC	71.02 ± 0.09^a^	75.83 ± 0.37^e^	84.57 ± 0.45^ab^	13.55 ± 0.39^ab^	5.10 ± 0.06^ab^
LF	69.54 ± 0.09^c^	76.88 ± 0.61^c^	84.74 ± 0.36^ab^	15.19 ± 0.42^a^	4.82 ± 0.11^bc^
LP	70.94 ± 0.94^ab^	78.01 ± 0.36^ab^	83.77 ± 1.05^ab^	12.83 ± 0.99^ab^	3.80 ± 0.15^e^
LC‐LF	69.97 ± 0.41^c^	76.71 ± 0.06 cd	83.45 ± 1.09^b^	13.48 ± 1.50^ab^	5.32 ± 0.17^a^
LC‐LP	71.16 ± 0.55^a^	77.19 ± 0.49^bc^	83.84 ± 1.23^ab^	12.68 ± 1.78^b^	4.15 ± 0.12^d^
LF‐LP	70.02 ± 0.21^bc^	75.95 ± 0.33^de^	85.01 ± 0.92^ab^	14.98 ± 0.71^ab^	4.55 ± 0.13^c^
LC‐LF‐LP	71.18 ± 0.27^a^	78.42 ± 0.48^a^	85.36 ± 0.26^a^	14.17 ± 0.17^ab^	5.40 ± 0.16^a^

Note

Simple corn flour (CF), *Lactobacillus Casei‐*treated corn flour (LC), *Lactobacillus Fermentum‐*treated corn flour (LF), *Lactobacillus Plantarum‐*treated corn flour (LP), *Lactobacillus Casei and Lactobacillus Fermentum‐*treated corn flour (LC‐LF), *Lactobacillus Casei* and *Lactobacillus Plantarum‐*treated corn flour (LC‐LP), *Lactobacillus Fermentum* and *Lactobacillus Plantarum‐*treated corn flour (LF‐LP), *Lactobacillus Casei*, *Lactobacillus Fermentum,* and *Lactobacillus Plantarum‐*treated corn flour (LC‐LF‐LP).

a‐f: Means in the same columns with different letters were significantly different (*p* < .05) from each other.

### 3.3 Rheological characterization of dough

**FIGURE 1:**
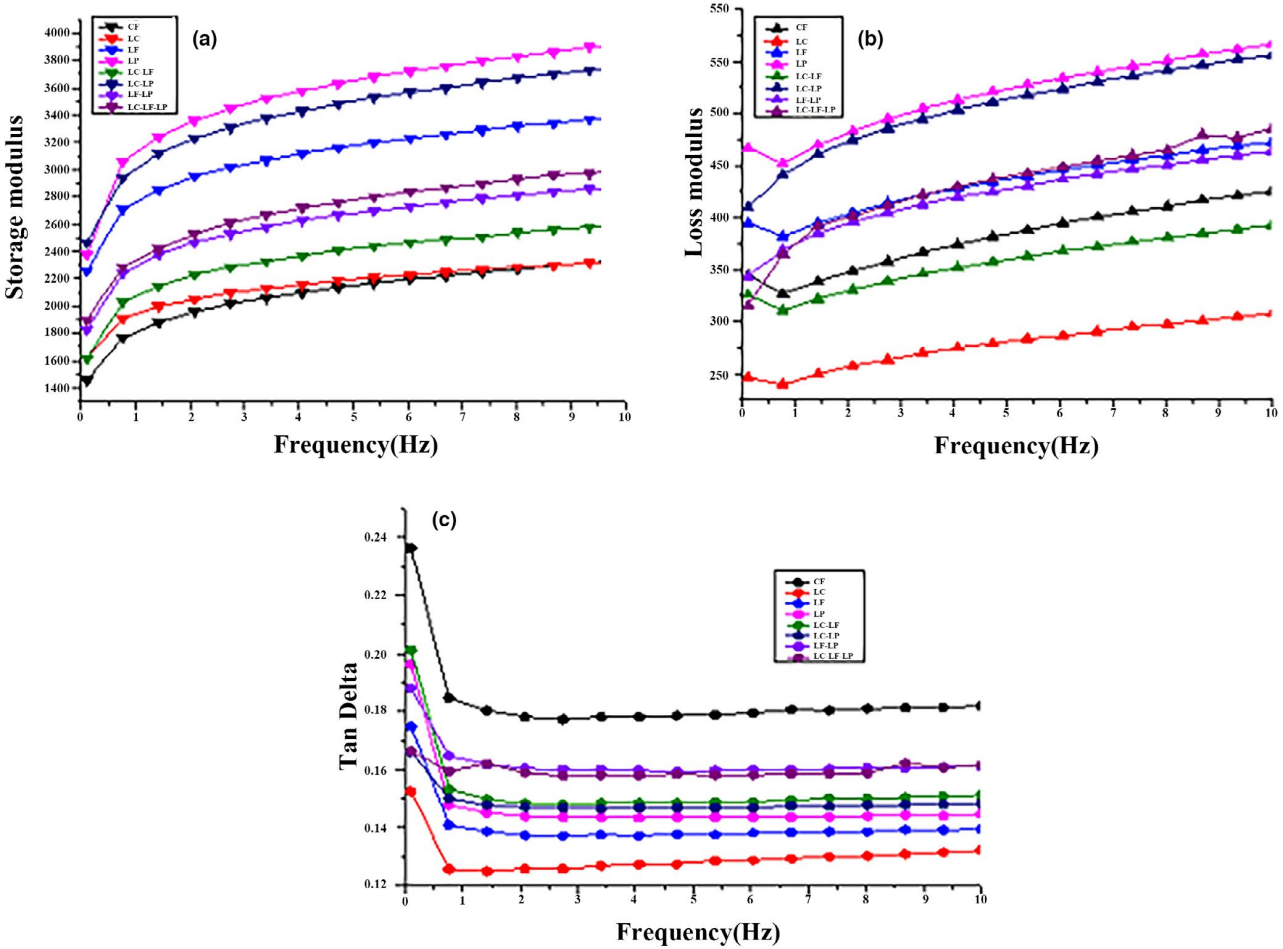
(a) Storage modulus [G’], (b) Loss modulus [G’’], (c) Tan Delta

The governing factor for proper dough manufacturing is its rheology. A frequency sweep test was conducted to study the quality and processability of dough ingredients. Comprehensive understanding of dough rheology is imperative for the production of a high quality end product (Letang et al., 1999). The storage modulus, loss modulus, and tan delta comprise the parameters for rheology of dough as shown in Figure 1. Both Storage modulus and loss modulus are directly proportional to frequency. Loss modulus is always inferior to storage modulus, indicating that dough was more elastic than viscous (Narsimhan, 1994). Bacterially fermented corn flour exhibited slightly higher storage modulus and loss modulus while a little lower tan delta than native corn flour. During gelatinization, amylose, amylopectin, and starch‐binding proteins and lipids leached out from the granules resulted in increased storage and loss modulus and less tan delta (Leon et al., 2006). LAB disrupt the physicochemical properties of flour and mainly hydrolyzed amylopectin into amylose which affect the rheological properties of flour (Yu et al., 2016). Enzymatic hydrolysis particularly the amylolytic enzymes led to the partial fragmentation of flour, that in turn affects the dough rheology.

### 3.4 Pasting properties of flour

Pasting parameters of native and bacterial fermented samples were summarized in Table 4. The whole modification significantly changes the overall shape of the visco profile by RVA. Bacterial fermentation results in reduced pasting parameters such as PV, PT, FV, breakdown, and setback. This could be accredited to the macromolecule degradation like starch, which considerably decreased during the process. The obtained results are in accordance with the findings of Ilowefah et al., (Ilowefah et al., 2015). They explicated the fact that acidification leads to breakage and more fragility of the starch granules. The pasting properties results are also closely consistent with the previous researchers, who stated that breakdown and setback of rice flour reduced after lactic acid fermentation (Yang & Tao, 2008). The key reason for these variations was the collapse of crystal structure in the starch granules during fermentation. Amylopectin swelling leads to formation of crystalline that results in more PV. Hence, breakdown and leaching of polymers along with decrease in PV is associated with polymerization and alteration in amylopectin chain (Chiang & Yeh, 2002). It is anticipated that viscoelastic properties are affected by some major moieties such as lipid, protein, and amylose contents. Amylopectin contributes in starch swelling however amylose is responsible for maintaining the swollen starch integrity and also suppresses the starch swelling (Jane et al., 1999). Higher amylose content is positively correlated to lower BV and PV. That is why, fermented flour swells more and degraded with substantial decrease in viscosity (Chung et al., 2011). On the other hand, the amylose leached from the system is rapidly aggregated when cooled. These amylose junction zones were responsible for the development of SV and FV (Barrera et al., 2013). Therefore, the extent of pasting properties of fermented flour was mainly (negatively/positively) correlated with the leaching of materials, damaged starch, and inner morphology. These aspects facilitated the interactions between molecules which involved in hydration.

**TABLE 4:** Effect of *L. Casei, L. Plantarum and L. Fermentum* on the RVA characterization of corn flour dough

Treatment	PV	TV	BV	FV	SV	PT	Pt
CF	1,783	1,038	745	1,834	796	75.85	4.40
LC	1,500	1,091	409	1,534	443	79.85	5.33
LF	1,301	983	318	1,348	365	80.70	5.26
LP	764	649	115	887	238	84.80	5.40
LC‐LF	1,403	1,059	344	1,462	403	81.90	5.13
LC‐LP	919	755	164	1,004	249	83.15	5.33
LF‐LP	1,294	985	309	1,472	487	79.90	5.13
LC‐LF‐LP	618	553	65	759	206	85.60	6.26

Note

Simple corn flour (CF), *Lactobacillus Casei‐*treated corn flour (LC), *Lactobacillus Fermentum‐*treated corn flour (LF), *Lactobacillus Plantarum‐*treated corn flour (LP), *Lactobacillus Casei and Lactobacillus Fermentum‐*treated corn flour (LC‐LF), *Lactobacillus Casei* and *Lactobacillus Plantarum‐*treated corn flour (LC‐LP), *Lactobacillus Fermentum* and *Lactobacillus Plantarum‐*treated corn flour (LF‐LP), *Lactobacillus Casei*, *Lactobacillus Fermentum,* and *Lactobacillus Plantarum‐*treated corn flour (LC‐LF‐LP).

### 3.5 Micromorphology of corn

**FIGURE 2:**
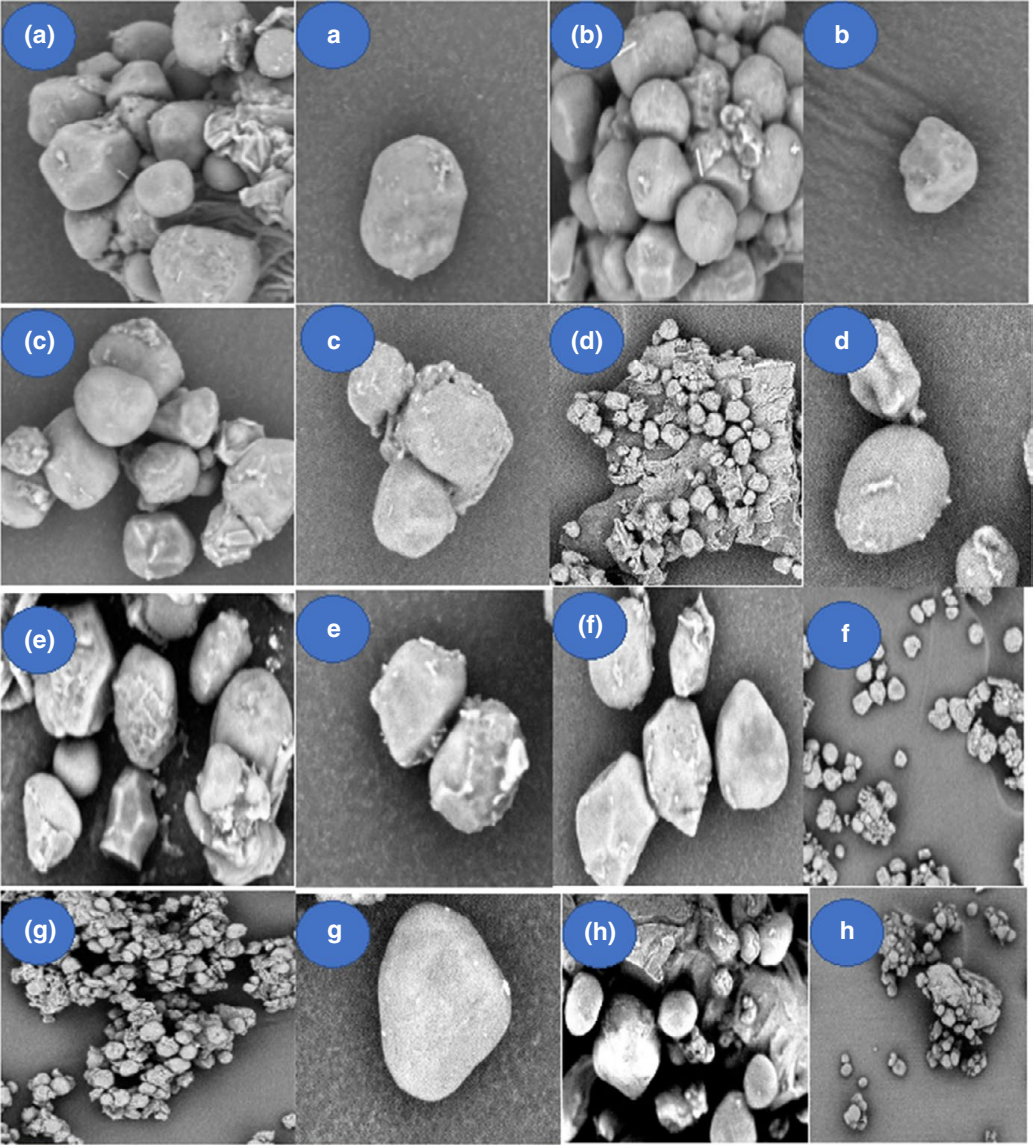
*SEM* photomicrographs of different samples: A/a LC, B/b LF, C/c LP, D/d LC‐LF, E/e LC‐LP, F/f LF‐LP, G/g LC‐LF‐LP, H/h CF. Micromorphology images of each sample were captured at the magnification of ×5000

*SEM* micrographs permit a direct observation of corn flour to explain the different properties of native and bacterial treated corn flour (Figure 2). Bacterial treated corn flour exhibits smaller particles having sharp and irregular edges, cracks, granule disruption, and more indentations while native flour retained smooth surface and robust structure (Di Stasio et al., 2007). Smaller and irregular particles in bacterial treated flour lead to increased water absorbing properties, lower water retention capacity, that eventually leads to compact dough with better rheological properties (Shrestha et al., 2015). Bacterial treatment directly affects and alters the flour morphological characteristics. The surface of treated samples with co‐culture and individual bacteria displayed more grooves and shallow indentations. It may be due to phase transformation and hydrolyzation that lead to suppressing and crumbling of granules and acquisition of irregular granules. Oh (Oh et al., 2008) reported irregular, cracked, and disrupted granules in modified corn starch compared to native starch.

## 4 CONCLUSIONS

The study concluded that bacterial treatments, individually and as a coculture modified the quality of flour and has potential applicability. These modifications positively impacted the structural, morphological, rheological, and thermal properties of corn dough. The dough morphology showed more grooves, irregular cracks, and shallow indentations that resulted in a better and more compact dough having good rheological properties. Lactic acid has potential industrial applications and hydrolyzed the flour leading to improved corn dough manufacturing. The results provided deeper insight and sufficient information regarding improvement in corn flour applicability due to bacterial incorporation and it may enhance the acquaintance about the upright revolution in the profile of corn dough and its potential usage in industry and homes.

## CONFLICT OF INTEREST

The authors declare no conflict of interest.

## ETHICAL APPROVAL

The manuscript does not involve any human or animal studies and it is according to the guidelines.

## Data Availability

The data that support the findings of this study are available from the corresponding author upon reasonable request.
